# Measurement of corneal thickness, optic nerve sheath diameter and retinal nerve fiber layer as potential new non-invasive methods in assessing a risk of cerebral edema in type 1 diabetes in children

**DOI:** 10.1007/s00592-018-1242-8

**Published:** 2018-10-16

**Authors:** Krzysztof Jeziorny, Anna Niwald, Agnieszka Moll, Katarzyna Piasecka, Aleksandra Pyziak-Skupien, Arleta Waszczykowska, Dobromiła Baranska, Beata Malachowska, Agnieszka Szadkowska, Wojciech Mlynarski, Agnieszka Zmyslowska

**Affiliations:** 1 Department of Pediatrics, Oncology, Hematology and Diabetology, Medical University of Lodz, Sporna Str. 36/50, 91-738 Lodz, Poland; 2 Outpatient Clinic of Pediatric Ophthalmology, Central Clinical Hospital, Lodz, Poland; 3 Department of Ophthalmology and Vision Rehabilitation, Medical University of Lodz, Lodz, Poland; 4 Department of Diagnostic Imaging, Polish Mother’s Memorial Hospital Research Institute, Lodz, Poland; 5 Department of Biostatistics and Translational Medicine, Medical University of Lodz, Lodz, Poland

**Keywords:** Type 1 diabetes, ONSD, CCT, RNFL, Cerebral edema

## Abstract

**Aims:**

Some patients with diabetic ketoacidosis develop cerebral edema (CE) in the course of type 1 diabetes mellitus (T1D), which may result in central nervous system disorders and high mortality. The imperfection of existing neuroimaging techniques for early recognition of CE forces us to search for the new and non-invasive methods. The aim of the study was to assess the usefulness of new methods (pachymetry, transorbital ultrasonography—USG, optical coherence tomography—OCT study) in the assessment of the risk of CE occurrence in children with newly diagnosed T1D.

**Methods:**

The study group included 50 children with newly diagnosed T1D, 54 patients with long-term T1D as a reference group and 40 children without glucose tolerance disorders as controls. In all subjects, a corneal thickness (CCT) index with pachymeter, optic nerve sheath diameter (ONSD) using transorbital USG and retinal nerve fiber layer (RNFL) during OCT study were measured and compared with selected clinical parameters of T1D.

**Results:**

In patients from a study group at onset of T1D, the higher CCT (*p* < 0.001) and ONSD (*p* < 0.001) values were observed as compared to the results obtained after 48 h of metabolic compensation. The ONSD correlated negatively with pH value (*r* = − 0.64; *p* < 0.001), BE (*r* = − 0.54, *p* < 0.001) and HCO^3−^ (*r* = − 0.50; *p* < 0.001). A positive correlation between RNFL and Na^+^ levels (*r* = 0.47; *p* < 0.005) was also observed.

**Conclusions:**

Transorbital USG and pachymetry may serve as the potential promising methods for the non-invasive assessment of the increased risk of development of CE in patients with T1D.

**Electronic supplementary material:**

The online version of this article (10.1007/s00592-018-1242-8) contains supplementary material, which is available to authorized users.

## Introduction

Diabetic ketoacidosis (DKA) is one of the most common acute complications of type 1 diabetes (T1D) in the pediatric population affecting 25–40% of the patients with newly diagnosed diabetes. About 0.5–1% of the patients with DKA develop cerebral edema (CE) which may result in temporary or permanent impairment of the central nervous system, with mortality rates up to 40–90% [1–3]. The precise pathological mechanism of CE development has not yet been fully understood. Currently, the most popular hypotheses are related to astrocyte edema caused by a decrease in extracellular volume (cellular edema) and breaking down of the blood–brain barrier caused by an increasing permeability of endothelial vessels (vasogenic edema) or an increase in proinflammatory cytokines or cerebral cell anoxia also associated with an increase in hypocapnia [2, 4–6]. The main risk factors of the development of CE in patients with DKA include: reduction of extracellular osmolarity, intensification of hypocapnia and metabolic acidosis, degree of dehydration, variability of sodium concentration during therapy, and iatrogenic errors mainly related to the supply of sodium bicarbonate to compensate acidosis [1, 4, 7].

At the moment, a diagnosis of CE is mainly based on a detailed clinical evaluation and neurological examination of patients. Neuroimaging studies such as magnetic resonance (MRI) or computed tomography (CT) as recommended by the International Society for Pediatric and Adolescent Diabetes (ISPAD) should be performed only after starting treatment and are intended to confirm the diagnosis and exclude acute conditions requiring neurosurgical intervention or anticoagulation treatment during such events as intracranial bleeding or cerebral thrombosis [8]. However, several studies showed that subclinical and asymptomatic CE may occur in many more patients than we earlier thought, and 40% of the patients with suspected CE do not exhibit any radiological findings during the neuroimaging studies [1, 2]. Moreover, brain MRI or CT imaging are associated with high economic costs including availability of study equipment and employment of highly educated medical staff, and also with a risk for a patient such as: exposure to ionizing radiation and sedation for the pediatric population [9].

Thus, the above-mentioned imperfection of neuroimaging as well as the invasive methods of direct measurement of increased intracranial pressure force us to find other indirect ways to exclude the potentially reversible state of life-threatening condition such as CE. These methods should be, however, widely available and performable before the treatment is started and the classical methods of neuroimaging are applied. Some recent studies on the potential use of transorbital ultrasonography (USG) to evaluate the risk of CE in the small groups of pediatric patients with DKA revealed encouraging results [10–12]. Other promising methods allowing an assessment of retinal nervous layer seem to be the optical coherent tomography (OCT) study [13] and a pachymetry evaluating corneal thickness [14, 15].

The aim of the study was to analyze the usefulness of pachymetry, transorbital USG and OCT study in the assessment of the risk of CE in children with newly diagnosed T1D.

## Materials and methods

Before initiating the study, the Bioethics Committee of the Medical University of Lodz, Poland approved the study protocol (RNN/20/15/KE) and all patients and/or their parents expressed written consent for participating in the study.

A study group consisted of 50 patients aged 5–18 years at the average age of 9.5 years (IQR 8.3–12.5) (F/M: 46%/54%) with newly diagnosed T1D as defined by WHO [16], with detected autoantibodies and reduced C peptide concentration [Me = 0.31 ng/ml (IQR 0.22–0.51)]. The median HbA1c level was 12.0% (IQR 10.8–13.2); [108 mmol/mol (IQR 95–121)].

All patients were treated with an intravenous insulin infusion. DKA was found in 22/50 (44%) of the patients and in 17/50 of the patients it was mild DKA (7.2 < pH < 7.3), whereas in 5/50 of children it was moderate or severe DKA (pH < 7.2). None of the patients from the study group received sodium bicarbonate (NaHCO_3_), mannitol nor 3% saline during the treatment.

All analyses in the study group were performed at two time points: at the onset of clinical diabetes diagnosis and after compensation of metabolic disturbances, i.e., after at least 48 h from the onset of clinically diagnosed T1D.

A reference group included 54 children aged 8–18 years at the average age of 13.2 years (IQR 10.6–16.2) (F/M: 57%/43%) admitted for a routine metabolic control assessment with T1D after at least two years of disease duration [Me = 5.5 years, (IQR 2.4–8.2)] without metabolic decompensation and with subcutaneous insulin treatment. The median HbA1c level was 7.3% (IQR 6.9–7.7); [56 mmol/mol (IQR 52–61)].

The control group consisted of 40 healthy children, aged from 6 to 18 years at the average age of 13.3 years (IQR 9.9–16.4) (F/M: 28%/72%) without any glucose tolerance disorders.

Patients aged < 5 years, with diagnosed hypertension as well as some serious diseases and changes in ocular morphology were excluded from the study.

An examination of corneal thickness was preceded by local anesthesia of the eyeball with a proxymetacaine preparation with a subsequent four-time evaluation of the central corneal thickness (CCT) index for each eye using the Tomey SP-100 pachymeter (Germany). All results were evaluated independently by an experienced ophthalmologist.

**Fig. 1:**
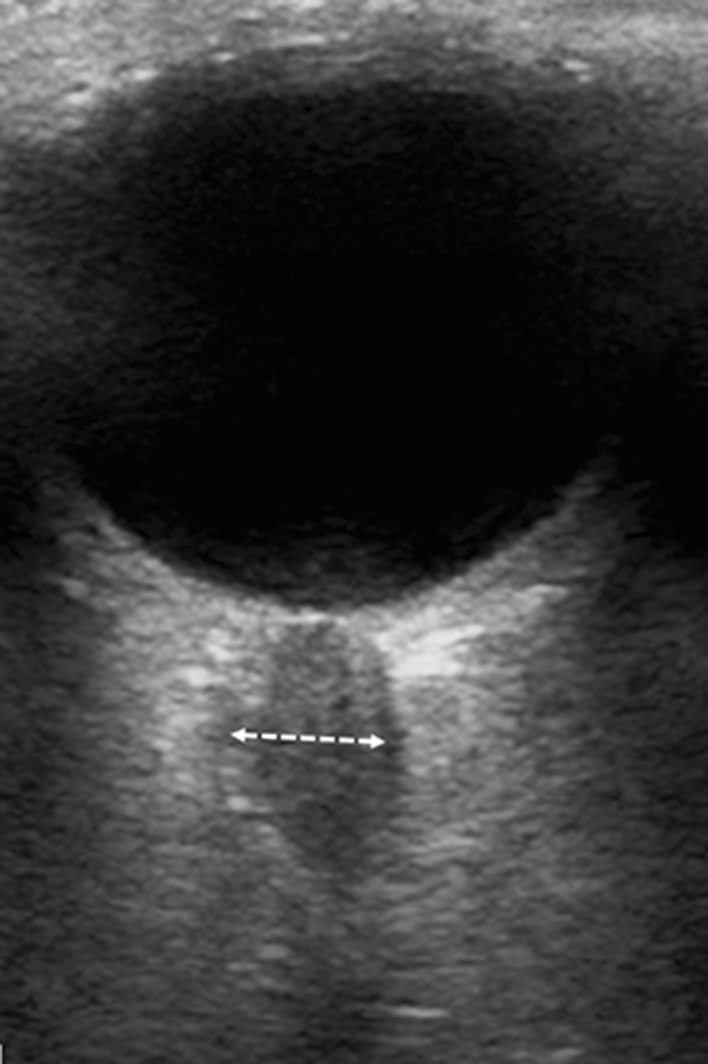
An ultrasonographic image of optic nerve sheath diameter (ONSD) measurement (marked by a dotted arrow)

During transorbital USG study, optic nerve sheath diameter (ONSD) was assessed in all subjects using a mobile Philips USG device (Holland) with a linear transducer (12–5 MHz) located on the patient’s closed eyelid of each eye in the transverse plane. The measurements were performed at a distance of 3 mm from the visual nerve disc (Fig. 1) and obtained results were verified using the RadiAnt DICOM Viewer software (Medixant, Poland). All studies were evaluated independently by an experienced radiologist.

During the OCT study, an assessment of retinal nerve fiber layer (RNFL) was performed using 3D Disc and 3D Macula scans with a 6 × 6 mm area (Topcon 3D OCT-2000 system, Japan) with the subjects in a resting position after an average of 15–20 min after mydriasis and evaluated independently by two experienced ophthalmologists.

Averages from measurements in both eyes were calculated and used for further analysis by comparing with clinical parameters including: presence and intensity of ketoacidosis (pH, BE, HCO^3−^), Na^+^ and K^+^ levels, serum and urine osmolality and urea concentration.

### Statistical analysis

Verification of the normality of distribution was carried out using the Kolmogorov–Smirnov and Lilliefors tests. The Wilcoxon test was used to compare the differences between parameters assessed at the time of T1D diagnosis and after 48 h. Comparison of the differences in parameters evaluated between the studied groups depending on normal distribution of data using a Student’s *t* test or a non-parametric Mann–Whitney test and a non-parametric Friedman ANOVA test was performed. For the correlation analysis, a Spearman correlation test was used. Categorical variables were presented as numbers with appropriate percentages and continuous variables as medians with interquartile range (IQR). Receiver operating characteristics (ROC) curves for increased CE risk model with calculating the area under the ROC (AUC) were evaluated. 95% confidence intervals (95% CI) were computed for AUCs. Results with *p* values < 0.05 were considered as statistically significant. Analyses were performed using Statistica 13.1 PL software (Statsoft, Tulsa, OK, USA).

## Results

In patients from a study group at the time of T1D diagnosis, higher CCT (*p* < 0.001) and ONSD (*p* < 0.001) values were observed as compared to the results obtained after 48 h of metabolic compensation. No differences were found for the RNFL values evaluated in the OCT study (*p* = 0.509) (Table 1).

**Table 1:** Comparison of studied parameters in a group of patients with newly diagnosed T1D at the onset of clinical diagnosis and after at least 48 h of metabolic compensation

Parameter	At T1D onset Median (IQR)	After > 48 h Median (IQR)	*p* level
CCT (µm)	**586 (563–616)**	**572 (550–590)**	< **0.001**
ONSD (cm)	**0.46 (0.43–0.47)**	**0.43 (0.41–0.45)**	< **0.001**
RNFL total thickness (µm)	102.0 (99.0–106.5)	101.5 (98.0–105.5)	0.509

*T1D* type 1 diabetes, *IQR* interquartile range, *CCT* central corneal thickness, *ONSD* optic nerve sheath diameter, *RNFL* retinal nerve fiber layer

*p* < 0.05 are indicated in bold

The CCT value in patients from a study group correlated negatively with CO_2_ (*r* = − 0.33; *p* = 0.032) and a trend with respect to a negative correlation between CCT and pH value was noted (*r* = − 0.26; *p* = 0.088).

The ONSD value correlated negatively with: pH value (*r* = − 0.64; *p* < 0.001), BE (*r* = − 0.54, *p* < 0.001) and HCO^3−^ (*r* = − 0.50; *p* < 0.001) and also a trend with respect to a correlation between ONSD and both the Na^+^ level (*r* = 0.26; *p* = 0.073) and CO_2_ (*r* = − 0.28; *p* = 0.064) concentrations was found.

A positive correlation between RNFL and Na^+^ levels (*r* = 0.47; *p* < 0.005) and a trend with respect to a positive correlation between RNFL and urea concentration (*r* = 0.32; *p* = 0.069) were also noticed. Other evaluated clinical parameters did not correlate with the values of the studied parameters (*p* > 0.09).

**Fig. 2:**
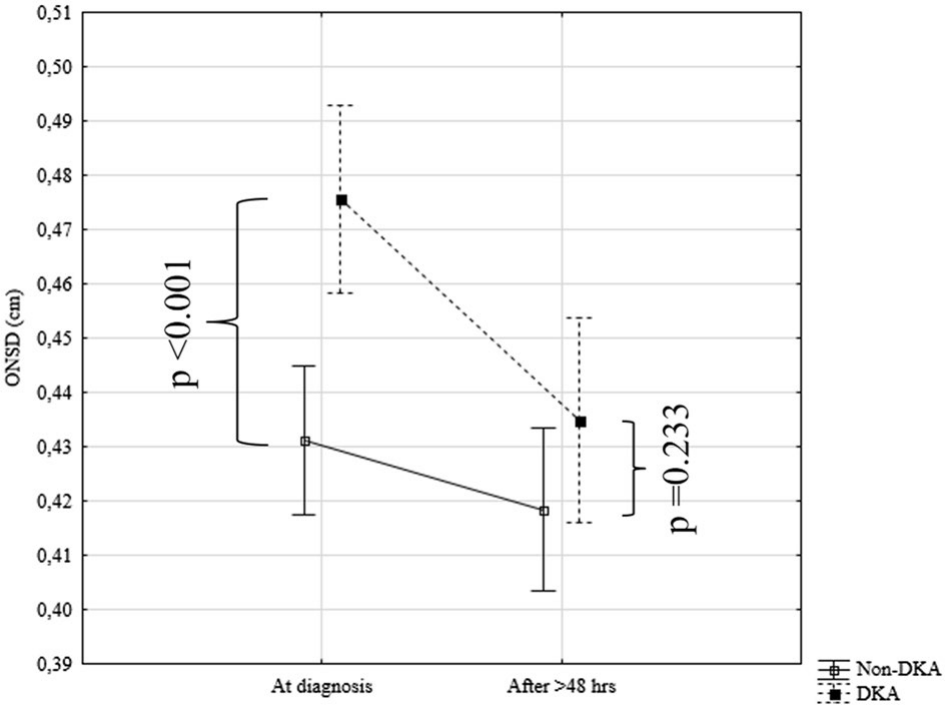
Changes in ONSD values in patients with and without ketoacidosis at clinical diagnosis of T1D and after at least 48 h of metabolic compensation

**Fig. 3:**
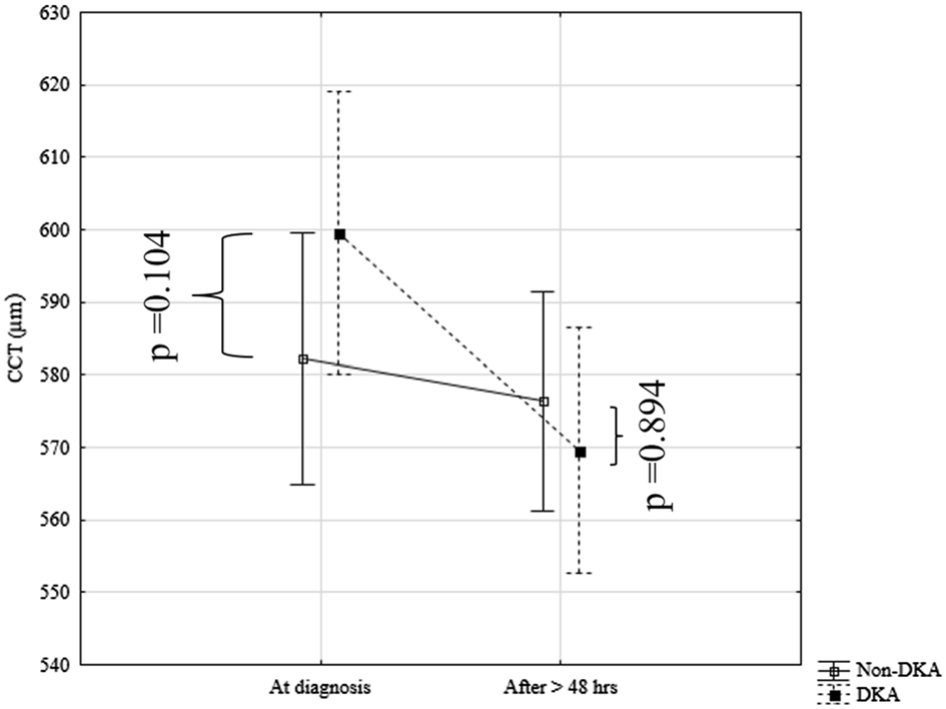
Changes in CCT values in patients with and without ketoacidosis at clinical diagnosis of T1D and after at least 48 h of metabolic compensation

In the patients from a study group with DKA in comparison to the non-DKA patients at the disease onset higher ONSD values (respectively: Me = 0.47 cm (IQR 0.47–0.48) vs. Me = 0.44 cm (IQR 0.41–0.46); *p* < 0.001) and a trend with respect to a higher CCT value (respectively: Me = 597 µm (IQR 566–625) vs. Me = 579 µm (IQR 550–594); *p* = 0.104) in a subgroup of patients with DKA were observed. After at least 48 h of metabolic compensation, no differences in the values of CCT (respectively: Me = 569 µm (IQR 552–591) vs. Me = 572 µm (IQR 548–591); *p* = 0.894) (Fig. 2) and in ONSD values between subgroups of patients with and without DKA [respectively: Me = 0.44 cm (IQR 0.43–0.45) vs. Me = 0.43 cm (IQR 0.39–0.46); *p* = 0.233] (Fig. 3) were noted.

For the RNFL value, no significant difference between DKA patients and non-DKA patients at the time of diagnosis (respectively: Me = 103 µm (IQR 100–105) vs. Me = 102 µm (IQR 99–109); *p* = 0.879) was observed. After metabolic disorder compensation, only a trend with respect to higher RNFL value in non-DKA patients was noted [Me = 100 µm (IQR 98–103) vs. Me = 103 µm (IQR 99–111); *p* = 0.093].

Comparing the values of the studied parameters between the study group of patients with a newly diagnosed T1D, taking into account both at the onset of disease and after at least 48 h of metabolic compensation, as well as the reference group of long-term T1D patients and healthy controls, differences in the values of CCT and ONSD parameters were observed. The CCT value was the highest at the onset of T1D in relation to other groups (*p* = 0.018) and decreased thereafter (> 48 h) but it was similar in relation to other groups (*p* = 0.161), whereas the ONSD value was the highest at T1D onset (*p* = 0.001) remaining thereafter the highest value in comparison to other groups (*p* = 0.035) (Table 2).

**Table 2:** Comparison of studied parameters between groups of patients with newly diagnosed T1D (at onset of disease and after at least 48 h of metabolic compensation), reference group (long-term T1D) and healthy controls

Parameter	Study group—at T1D onset Median (IQR)	Reference group Median (IQR)	Control group Median (IQR)	*p* level
CCT (µm)	**586 (563–616)**	**580 (556–602)**	**566 (538–587)**	**0.018**
ONSD (cm)	**0.46 (0.43–0.47)**	**0.40 (0.41–0.44)**	**0.41 (0.39–0.42)**	**0.001**
RNFL total thickness (µm)	102.0 (99.0–106.5)	102.7 (98.2–107.2)	101.0 (91.2–108.2)	0.301

Parameter	Study group—after > 48 h Median (IQR)	Reference group Median (IQR)	Control group Median (IQR)	*p* level
CCT (µm)	572 (550–590)	580 (556–602)	566 (538–587)	0.161
ONSD (cm)	**0.43 (0.41–0.45)**	**0.40 (0.41–0.44)**	**0.41 (0.39–0.42)**	**0.035**
RNFL total thickness (µm)	101.5 (98.0–105.5)	102.7 (98.2–107.2)	101.0 (91.2–108.2)	0.308

*T1D* type 1 diabetes, *IQR* interquartile range, *CCT* central corneal thickness, *ONSD* optic nerve sheath diameter, *RNFL* retinal nerve fiber layer

*p* < 0.05 are indicated in bold

After analyzing the whole diabetic population in comparison to the healthy controls, the group of T1D patients had higher CCT [Me = 581 µm (IQR 557–604) vs. Me = 566 µm (IQR 538–587); *p* = 0.019] and ONSD values [Me = 0.44 cm (IQR 0.41–0.46) vs. Me = 0.41 cm (IQR 0.39–0.42); *p* < 0.001] with no differences for RNFL values (*p* = 0.302).

In addition, the ROC curves and AUCs for the analyzed parameters dividing the whole group of T1D patients into patients without DKA (lower CE risk) vs. with DKA (increased CE risk) were evaluated. For CCT, the AUC of the model was 0.64 (95% CI 0.50–0.78). The model’s sensitivity and specificity equaled 63.2% and 70.5%, respectively (*p* = 0.048) (Figure Suppl. 1). For ONSD, the AUC was 0.91 (95% CI 0.85–0.98) with the model’s sensitivity and specificity of 83.3% and 87.8%, respectively (*p* < 0.0001) (Figure Suppl. 2). No significant predictive values for RNFL were obtained (*p* = 0.784; AUC 0.52).

## Discussion

For the first time, the usefulness of three different methods of transorbital USG, pachymetry and OCT in a detection of increased CE risk in pediatric patients with a newly diagnosed T1D with various degrees of metabolic decompensation was evaluated. Furthermore, the ROC curves and AUCs for the CCT, ONSD and RNFL parameters were established, dividing diabetic patients into those without DKA (lower CE risk) and with DKA (increased CE risk). For CCT and ONSD parameters, we were able to calculate the prediction values, whereas it was not relevant for RNFL value. It is worth noting that the presence and severity of acidosis in children with a newly diagnosed T1D is one of the important factors in the risk of CE occurrence [1, 7].

In the presented study, higher CCT values were observed at the onset of T1D in comparison to the values found in the same patients after 48 h of metabolic compensation of diabetes and CCT values correlated negatively with gasometry parameters. Other studies conducted in the group of healthy children and adult patients as well as patients with diabetes showed statistically significant differences in CCT thickness related to hyperglycemia, metabolic control of diabetes and disease duration. In patients with a higher percentage of HbA1c [17] and longer duration of the disease, higher CCT values were observed in comparison with control groups [14, 15, 18]. All authors emphasize higher CCT dimensions in patients with T1D compared to subjects without glucose tolerance disorders [14, 15, 17–20].

To date, no studies to assess the usefulness of pachymetry in children with a newly diagnosed T1D in relation to their metabolic decompensation have been performed. The precise pathological mechanism of corneal lesions has not been fully explained. However, the causes of these differences include both polyol tract, (i.e., glucose converts into hydrophilic sorbitol in endothelial cells during hyperglycemia and therefore an increase is observed in CCT) [15, 21] and a decrease in activity of endothelial Na^+^/K^+^ ATPase of the cornea [14].

In our study, the best method to assess the increased risk of CE in children with ketoacidosis in the course of a newly diagnosed T1D seems to be an ultrasound examination of the eyeball. Due to the connection between the subarachnoid space surrounding the sheath and the visual junction reservoir which is a part of the central nervous system, an increase in intracranial pressure results in relocation of the cerebrospinal fluid and dilatation of the ONSD dimensions [22].

So far, there have been single reports in the literature on the use of ONDS in the pediatric diabetic population which gives equivocal results. Bergmann et al. did not note differences in ONSD between the studied groups with long-term T1D with DKA vs. non-DKA and hyperglycemic T1D, but they observed mean ONSD values in the group of T1D above the references range [12]. Hansen et al. observed a tendency to decrease the dimensions of ONSD after compensation of metabolic disorders, but the study was carried out on a low number of patients [11]. Szmygel et al. noticed a significant reduction in the ONSD dimension in non-DKA patients and a tendency in the DKA group of patients with T1D [10].

In our study, we observed that patients with the DKA vs. non-DKA were characterized by significantly higher ONSD values, with a lack of differences after compensation of acute metabolic disturbances. ONSD values correlated negatively with many gasometry parameters and a tendency towards positive correlation with Na^+^ level was also observed. Moreover, comparing the whole group of patients with T1D with the reference group, we have found significantly higher ONSD values, as in the research of Bergman et al. [12].

In many studies, an increased intracranial pressure and an indirect risk of CE were considered to be the result of a ONSD value of 4.5–5 mm [22–26]. The ONSD measurement was characterized by a high level of repetitiveness and low result variability by various researchers [9, 23, 27]. This method is quick and easy to use with its high specificity and sensitivity compared to MRI [9, 28]. This non-invasive method of intracranial pressure measurement is currently used in patients with craniocerebral trauma in many emergency rooms and ICU wards [24, 29].

In the presented study, the usefulness of the latter method in the assessment of the increased CE risk in the OCT study was evaluated. Thus, RNFL values have not been shown to be sufficiently useful for the CE risk assessment. The reduced value of RNFL assessment during the OCT study was already defined as a marker of many neurodegenerative disorders, including the syndromic forms of insulin-dependent monogenic diabetes coexisting with neurodegeneration [13, 30–32]. However, it should be pointed that the low availability of devices and difficulties in obtaining results caused by cooperation with small children (visual fixation necessary) and/or being in severe clinical condition can limit the usage of this method as a routine method for the detection of CE risk.

Our study has several limitations. The aim of our study was to analyze the usefulness of new methods such as pachymetry, transorbital USG and OCT study in determining only the increased risk of CE. Therefore, only a routine neurological evaluation in patients was performed with no detailed evaluation according to a Glasgow scale and no classical methods of neuroimaging (brain MRI and CT) for the comparison of the obtained results were performed. Next, because of the exclusion of a study group of children under 5 years of age with a newly diagnosed T1D due to a lack of cooperation during the OCT study, the study has only preliminary character and further studies in a larger group of a newly diagnosed pediatric T1D patients are needed to confirm the real usefulness of the above proposed methods.

In summary, it seems that transorbital ultrasound and pachymetry may serve as the potential promising methods for the non-invasive assessment of the increased risk of development of cerebral edema in pediatric patients with type 1 diabetes. However, the study is only preliminary and further studies are needed to confirm their effectiveness and the possibility of supplementing them with the classical methods of neuroimaging as the future diagnostic standards for children with type 1 diabetes.

## Electronic supplementary material

Below is the link to the electronic supplementary material.

Supplementary material 1 (DOCX 33 KB)
