# Supplementary material for: Measurement of corneal thickness, optic nerve sheath diameter and retinal nerve fiber layer as potential new non-invasive methods in assessing a risk of cerebral edema in type 1 diabetes in children
Source: Acta Diabetol. 2018 Oct 16;55(12):1295–301. doi: 10.1007/s00592-018-1242-8 (PMC6244862; doi:10.1007/s00592-018-1242-8)
Supplement: Supplementary file 1 — Supplementary material 1 (DOCX 33 KB) [file 592_2018_1242_MOESM1_ESM.docx]

Figure Suppl. 1. ROC curve for the CCT value predicting the increased CE risk model based on DKA presence (AUC= 0.64 (95%CI 0.50-0.78)).

Figure Suppl. 2. ROC curve for the ONSD value predicting the increased CE risk model based on DKA presence (AUC= 0.91 (95%CI 0.85-0.98)).
